# Microstructural Changes during Degradation of Biobased Poly(4-hydroxybutyrate) Sutures

**DOI:** 10.3390/polym12092024

**Published:** 2020-09-04

**Authors:** Ina Keridou, Lourdes Franco, Luis J. del Valle, Juan C. Martínez, Lutz Funk, Pau Turon, Jordi Puiggalí

**Affiliations:** 1 Departament d’Enginyeria Química, Universitat Politècnica de Catalunya, Escola d’Enginyeria de Barcelona Est-EEBE, c/Eduard Maristany 10-14, 08019 Barcelona, Spain; ina.keridou@gmail.com (I.K.); lourdes.franco@upc.edu (L.F.); luis.javier.del.valle@upc.edu (L.J.d.V.); 2 Barcelona Research Center for Multiscale Science and Engineering, Universitat Politècnica de Catalunya, Escola d’Enginyeria de Barcelona Est-EEBE, 08019 Barcelona, Spain; 3 ALBA Synchrotron Light Facility, Carrer de la Llum 2-26, Cerdanyola del Vallès, 08290 Barcelona, Spain; guilmar@cells.es; 4 B. Braun Surgical, S.A.U. Carretera de Terrassa 121, Rubí, 08191 Barcelona, Spain; lutz.funk@bbraun.com (L.F.); pau.turon@bbraun.com (P.T.)

**Keywords:** poly(4-hydroxybutyrate), hydrolytic degradation, enzymatic degradation, sutures, films, microstructure, lamellar thickness, small angle X-ray scattering

## Abstract

Fibers of poly(4-hydroxybutyrate) (P4HB) have been submitted to both hydrolytic and enzymatic degradation media in order to generate samples with different types and degrees of chain breakage. Random chain hydrolysis is clearly enhanced by varying temperatures from 37 to 55 °C and is slightly dependent on the pH of the medium. Enzymatic attack is a surface erosion process with significant solubilization as a consequence of a preferent stepwise degradation. Small angle X-ray diffraction studies revealed a peculiar supramolecular structure with two different types of lamellar stacks. These were caused by the distinct shear stresses that the core and the shell of the fiber suffered during the severe annealing process. External lamellae were characterized by surfaces tilted 45° with respect to the stretching direction and a higher thickness, while the inner lamellae were more imperfect and had their surfaces perpendicularly oriented to the fiber axis. In all cases, WAXD data indicated that the chain molecular axis was aligned with the fiber axis and molecules were arranged according to a single orthorhombic structure. A gradual change of the microstructure was observed as a function of the progress of hydrolysis while changes were not evident under an enzymatic attack. Hydrolysis mainly affected the inner lamellar stacks as revealed by the direct SAXS patterns and the analysis of correlation functions. Both lamellar crystalline and amorphous thicknesses slightly increased as well as the electronic contrast between amorphous and crystalline regions. Thermal treatments of samples exposed to the hydrolytic media revealed microstructural changes caused by degradation, with the inner lamellae being those that melted faster.

## 1. Introduction

Synthetic bioabsorbable sutures have been commercialized since the early 1970s when braided polyglycolide sutures were developed [1]. Since then, different homopolymers and copolymers have been employed in order to provide a controlled degradation rate and a good fit with the required function as a temporary wound support. Although initial sutures were processed in a braided form to reduce stiffness and facilitate manipulation, the use of resorbable monofilament forms was since the 1980s when polydioxanone sutures were developed [2]. Advantages of this form involve the reduction of problems associated to tissue drag and the decrease of infection risk derived from a capillary effect. In general, traditional monofilament sutures have a fast or medium degradation rate, with a long term decomposition profile having been developed more recently. Specifically, MonoMax^®^ has been commercialized in 2009 for abdominal wall repair applications [3]. This suture is based on poly(4-hydroxybutyrate) (P4HB) and is currently probably the most pliable monofilament suture. Specific mechanical properties of P4HB are 50 MPa, 70 MPa, and 1000% for tensile strength, tensile modulus, and elongation at break [4]. The polymer is fully biocompatible since its degradation leads to 4-hydroxybutyrate, which is a molecule resulting from the metabolism of 4-aminobutyrate (GABA). The degradation process of the polymer P4HB in the human body is initiated by hydrolysis caused by the water diffused into the polymer bulk [5], but enzymes such as lipases are also able to promote a surface attack [5,6].

Poly(hydroxyalkanoate)s (PHA)s constitute a big family of polyesters that show common properties such as biocompatibility, biodegradability, and non-toxicity [7]. These properties together with a great elasticity justify the increasing use of P4HB in different biomedical applications [8]. In fact, P4HB is the only PHA that has been approved by the FDA (2007) for biomedical uses. In addition to MonoMax (i.e., a long term bioresorbable suture), TephaFLEX, BioFiber, Phasix, and GalaFLEX are other commercial P4HB based materials that are employed in medical devices such as abdominal wall closure materials, tendon repair scaffolds, hernia repair meshes, and reconstructive surgery materials [6,9,10,11].

P4HB sutures and implants in general have advantages derived from the gradual loss of mechanical properties and the gradual release of degradation products into the blood that is in contrast with the behavior of the firstly employed polyglycolide materials [12].

Commercial P4HB is obtained using fermentation processes. Chemical synthesis is disfavored because of the low molecular weight samples (i.e., around 5000 g/mol) that have been attained in most of the studied processes [13,14]. Formation of γ-butyrolactone rings is kinetically favored with respect to the polymer chain extension and consequently ring-opening polymerization is only feasible under highly expensive high-pressure processes, which lead to moderate molecular weight around 50,000 g/mol [14]. The biosynthesis of P4HB is rather complex since typical bacteria (e.g., *Ralstonia eutropha* that was the first one employed) also incorporate 3-hydroxybutyrate units despite employing nutrient media based only in 4-hydroxybutyrate and γ-butyrolactone [15]. Currently, the P4HB homopolymer is mainly obtained from engineered *E. coli* K12 [16] since this transgenic microorganism can produce the P4HB homopolymer even from inexpensive carbon sources such as glucose or lactose.

P4HB is a semicrystalline polymer able to crystallize as single lamellar crystals and defined by an orthorhombic structure (*a* = 0.775 nm, *b* = 0.477 nm, and *c* (fiber axis) = 1.199 nm) as deduced from electron and X-ray diffraction patterns [17,18,19]. This structure is defined by an antiparallel arrangement of molecular chains that adopt a slightly distorted all-trans conformation.

Monofilament P4HB threads are submitted to extensive annealing processes under mechanical stress and temperature before commercialization. This treatment has repercussions on the melting behaviour due to the reorganization of constitutive crystals. Thus, the melting temperature becomes close to 72 °C after annealing, a value that contrasts with the temperature of 58 °C determined for melt crystallized samples [20,21]. Stretching of P4HB leads to a significant increase of its rigidity while flexibility is maintained. This is a distinctive feature with respect to those of other common biodegradable polyesters such as polyglycolide and polylactide [6], which become brittle under stress and consequently cannot be submitted to similar processes of alignment. Therefore, P4HB can display a particular microstructure that should be characterized by a high orientation of molecular chains along the stretching direction and a compact stacking of constitutive lamellae.

Microstructure and crystalline morphology of materials are crucial factors that have an influence on their degradability. It is well known that degradation proceeds through the amorphous regions and consequently the specific arrangement of spherulites (melt crystallized samples) and lamellar stacks (oriented and annealed fibers) are meaningful. Degradation conditions (e.g., pH of hydrolytic media or the presence of enzymes) affect the microstructure on the material and may lead to distinctive morphological features as consequence of preferential attack to the surface or the bulk, and in this case on interlamellar stacks or interfibrillar domains [22]. An assessment of the effect of degradation on the microstructure of stretched P4HB fibers was the main goal of the work reported here due to the peculiar and highly oriented molecular arrangement that can be attained with this high molecular weight and flexible polymer. Results should be interesting to progress on the comprehension of the relationships between crystalline morphology and degradability.

## 2. Materials and Methods

Commercially available sutures of P4HB (Monomax^®^ USP 1) were kindly supplied by B. Braun Surgical S.A.U. Weight and number average molecular weights of Monomax^®^ samples were 215,000 and 68,000 g/mol, as determined by GPC.

*Pseudomona cepacia* and *Rhizopus oryzae* enzymes with a specific activity of 40.0 and 55.7 U/mg solid, respectively, were obtained from Sigma-Aldrich (Madrid, Spain). All reagents, citric acid, phosphoric acid, chloride acid, boric acid, sodium hydroxide, sodium azide, and chloroform (CHCl_3_) were supplied by Fisher Chemical (Hampton, NH, USA).

### 2.1. Hydrolytic and Enzymatic Degradation

In vitro hydrolytic degradation studies were directly carried out with commercial sutures (USP 1) with 1 cm long fragments. For the sake of completeness, melt pressed films (5 bars, 60 °C) with dimensions of 1 cm × 1 cm × 150 μm were also evaluated. Assays were carried out at 37 and 55 °C at different pH values of 3, 7, and 10 using the Universal buffer (citrate-phosphate-borate/HCl) solution [23]. This was prepared by mixing 20 mL of the stock solution with *x* mL of 0.1 M HCl and distilled water up to 100 mL. The stock solution (1 L) contained 100 mL of citric acid, 100 mL of phosphoric acid, 3.54 g of boric acid, and 343 mL of 1 M NaOH. Therefore, the buffers of pH 3, pH 7, and pH 10 values were obtained by mixing 20 mL of the stock solution and 56.9, 32.9, and 18.1 mL of 0.1 M HCl, respectively. Samples were kept under orbital shaking in bottles filled with 50 mL of the degradation medium and sodium azide (0.03 wt%) to prevent microbial growth for selected exposure times. The samples were then thoroughly rinsed with distilled water, dried to constant weight at reduced pressure, and stored over P_4_O_10_ before analysis. Weight retention was evaluated during degradation as well as the changes on molecular weight. Degradation studies were performed in triplicate and the given data correspond to the average values.

Enzymatic degradation studies were performed with both sutures and melt pressed films having the above indicated geometry. All samples were exposed to 1 mL of phosphate buffered saline (PBS) (pH 7.4) containing the determined enzyme alongside with sodium azide (0.03 % *w*/*v*). These solutions were renewed every 48 h to prevent enzymatic activity loss. Samples were kept at 37 °C in an orbital shaker at 80 rpm. Samples were taken from the media at determined times, washed three times with Milli-Q water, and dried in an oven at 37 °C for 24 h to determine the dry weight. All the experiments were conducted in triplicate. The degraded samples were carbon coated and observed in SEM with an accelerating voltage of 10 kV.

### 2.2. Measurements

The molecular weight was estimated by size exclusion chromatography (GPC) using a liquid chromatograph (Shimadzu, model LC-8A, Tokyo, Japan) equipped with an Empower computer program (Waters, Milford, MA, USA). A PL HFIP gel column (Polymer Lab) and a refractive index detector (Shimadzu RID-10A, Tokyo, Japan) were employed. The polymer was dissolved and eluted in 1,1,1,3,3,3-hexafluoroisopropanol (HFIP) containing CF_3_COONa (0.05 M) at a flow rate of 0.5 mL/min (injected volume 100 μL, sample concentration 2.0 mg/mL). The number and weight average molecular weights were calculated using polymethyl methacrylate standards.

^1^H-NMR spectra were acquired with a Bruker NMR Ascend 400 spectrometer (Bilerica, MA, USA) operating at 400 MHz. Chemical shifts were calibrated using tetramethylsilane as an internal standard. Deuterated chloroform was used as a solvent.

Calorimetric data were obtained by differential scanning calorimetry with a TA Instruments Q100 series (NewCasttle, DE, USA) equipped with a refrigerated cooling system (RCS) operating at temperatures from −50 to 150 °C. Calibration was performed with indium. Experiments based on heating runs at 10 °C/min were conducted under a flow of dry nitrogen with a sample weight of approximately 5 mg.

WAXD and SAXS data were obtained at the NCD beamline (BL11) of the ALBA synchrotron facility (Cerdanyola del Vallès, Barcelona, Spain), by using a wavelength of 0.100 nm. A WAXD LX255-HS detector from Rayonix and an ImXPAD S1400 photon counting detector were employed. Polymer samples were confined between Kapton films. WAXD and SAXS diffraction patterns were calibrated with Cr_2_O_3_ and silver behenate (AgBh), respectively. The correlation function and the corresponding parameters were calculated with the CORFUNC software for Fibre Diffraction/Non-Crystalline Diffraction provided by the Collaborative Computational Project 13.

The calculations of the parameters such as *L* or the angle that forms the lamellae with the fibre axis in the SAXS patterns have been carried out by means of a Python based software developed by the authors. It calculates the distance from the direct beam position to the center of a 2D elliptical Gaussian function fitted in a user defined ROI. If the center of the Gaussian falls outside the ROI, then an azimuthal integrational [24] is done in the ROI to fit a 1D Gaussian. This distance in pixels is converted to *q* vector units by means of a calibration file that was generated from a well-known standard, (i.e., silver behenate (AgBh)). As the SAXS patterns have some symmetry, the calculation are replicated to its specular reflection on equatorial or meridional axes depending on the case. Finally, an average of the calculated values are shown as a result. Analogously, a calculation for the angles is done.

Scanning electron micrographs were taken using a Phenom XL Desktop SEM equipment (Waltham, MA, USA). Degraded films were mounted on a double-sided adhesive carbon disc and were sputter-coated with a thin layer of carbon to prevent sample charging problems using a K950X Turbo Evaporator (West Sussex, UK). All samples were observed at an accelerating voltage of 10 kV.

### 2.3. Statistical Analyses

Values were averaged and graphically represented together with their respective standard deviations. Statistical analysis was performed by the one-way ANOVA test to compare the means of all groups, and then Tukey’s test was applied to determine a statistically significant difference between the two groups. The test confidence level was set at 95% (*p*  <  0.05).

## 3. Results and Discussion

### 3.1. Hydrolytic and Enzymatic Degradation of P4HB Sutures

Hydrolytic degradation of commercial P4HB sutures was evaluated through weight loss and molecular weight measurements using media of three different pH values (i.e., acidic, neutral, and basic) and two temperatures (i.e., 37 and 55 °C that are associated to physiological conditions and the higher available temperature before starting fusion, respectively).

Weight loss (*W_l_*) of the specimens was determined through Equation 1 where *W_d_* is the sample weight after degradation and *W_0_* is the initial sample weight:

*W_l_* = 100 × (*W_0_* − *W_d_*)/*W_o_* (1)

**Figure 1:**
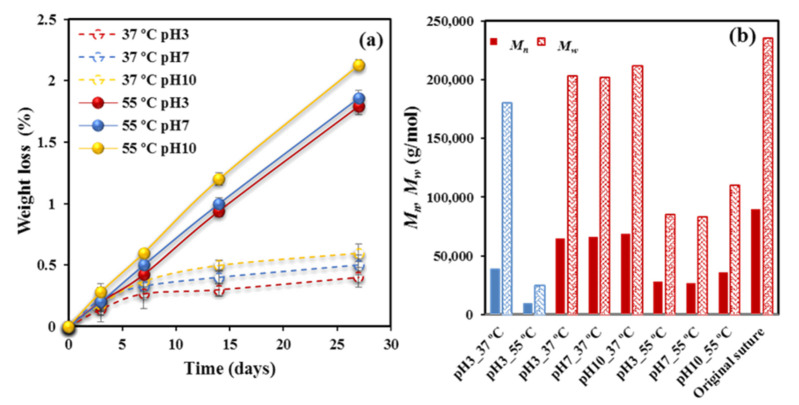
(**a**) Weight loss versus exposure time for P4HB sutures exposed to hydrolytic media of pH values of 3, 7, and 10 at temperatures of 55 °C (solid lines) and 37 °C (dashed lines). (**b**) *M_n_* (full bars) and *M_w_* (weave bars) molecular weights of the initial suture and after exposure for 27 days to the indicated media. For the sake of completeness, the results obtained from a melt pressed film are also shown (blue bars).

Figure 1a clearly reveals that scarce soluble fragments were produced during degradation since a loss of only 2.1–1.8% was achieved after 27 days of exposure to the media at 55 °C.

A slightly higher loss was detected for experiments performed at the pH 10 basic medium since fragments having the neutralized carboxylate terminal groups coming from P4HB degradation should have a higher water solubility than those ending with carboxylic acid groups. Exposure to the 37 °C medium caused a minimum weight loss (0.6–0.4%), mainly associated to the first days and which probably corresponded to the solubilization of minor additives as typical colorant molecules.

Therefore, evidences of degradation were only found through GPC measurements. *M_n_* and *M_w_* data after 27 days of exposure to the indicated media and temperature are depicted in Figure 1b for the studied sutures and a representative film was exposed to pH 3. Three points merit attention: (a) Degradation is highly significant at 55 °C, decreasing, for example, *M_w_* from 235,000 g/mol to a minimum value of 83,000 g/mol. On the contrary, a scarce variation was found for samples degraded at 37 °C. (b) The pH of the medium has a moderate influence on degradability, which specifically becomes slightly enhanced in the acidic condition. This feature confirms the above indicated association between weight loss and solubility. Note that the reaction may also be base-catalyzed, although the given results pointed out to an apparent acid-catalysis. (c) Degradability is highly dependent on the crystallinity and morphology of exposed samples. Note the high variation between *M_w_* values of annealed sutures and melt pressed films after exposure to pH 3 media at 55 °C (i.e., 83,000 g/mol with respect to 25,000 g/mol). Even a remarkable difference is found at 37 °C (i.e., 200,000 g/mol with respect to 180,000 g/mol).

**Figure 2:**
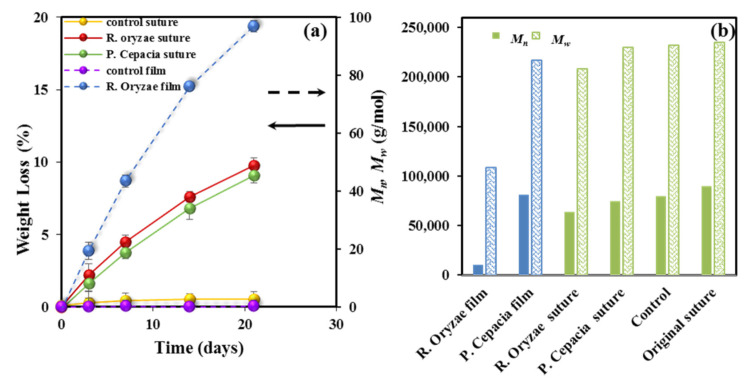
(**a**) Weight loss versus exposure time for P4HB sutures exposed to the indicated enzymatic media. Results are also plotted for the control and a melt pressed film (right vertical axis, dashed arrow) exposed to the *Rhizopus oryzae* medium for 21 days. (**b**) *M_n_* (full bars) and *M_w_* (weave bars) molecular weights of the initial suture, the control and sutures after exposure for 21 days to the indicated enzymatic media. For the sake of completeness, the results obtained from a melt pressed film are also depicted (blue bars).

Degradation in the presence of two different lipases which are able to promote hydrolysis of the ester bonds of P4HB has been evaluated. Results are quite different from those attained with the hydrolytic degradation due to high efficacy of the enzymatic attack and also to its characteristic erosion mechanism that contrasts with the bulk process associated to the hydrolytic process. Figure 2a, shows the evolution of weight loss during exposure to both enzymatic media and to an aqueous medium at 37 °C and pH 7 used as a control. *Rhizopus oryzae* seems more effective that *Pseudomonas cepacia* enzyme, but both lead to a significant weight loss (i.e., 9–10%) that is clearly higher than observed for the control. It is clear that the enzymatic attack should produce small fragments probably as a consequence of a stepwise chain scission from the terminal groups that contrasts with the random bond cleavage expected from the bulk degradation.

Figure 2a also displays the results attained for a melt pressed film, which reveals again the decisive influence of crystallinity and the annealed morphology on degradability. Figure 2b shows the impact of enzymatic degradation on the molecular weight, which is summarized as follows: (a) A progressive decrease of molecular weight with the exposure time is observed for both enzymatic media. (b) Hydrolytic degradation seems negligible under the low temperature conditions. (c) The enzymatic attack is more effective than hydrolysis at high temperature (e.g., *M_n_* values of 64,000 and 28,000 g/mol were determined after 21 days of exposure to the *Rhizopus oryzae* medium and to the aqueous pH 3 medium at 55 °C after 27 days, respectively). (d) Enzymatic attack is less effective on the annealed and highly crystalline sutures than on the melt pressed films, demonstrating again the difficulty of enzymes to erode the constitutive crystals and a limited activity towards amorphous regions, including folding lamellar surfaces.

### 3.2. Influence of Degradation on Thermal Properties

Sutures are submitted to a set of thermal and stretching treatment processes in order to improve their mechanical performance. This treatment has a significant influence on crystallinity but also on morphological features, such as the thickness of the constitutive lamellae. As described in the preceding section, crystallinity plays a determinant role on the degradability of samples, but it is also evident that thermal properties will be affected, as well as the variation of crystalline morphological parameters during degradation processes.

**Figure 3:**
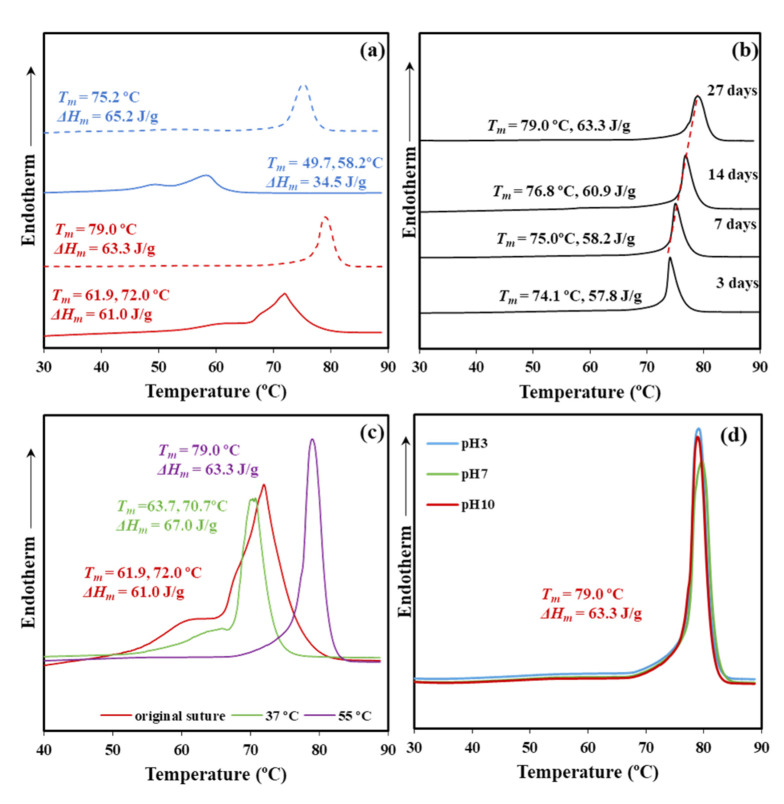
(**a**) DSC heating scans (10 °C/min) corresponding to a melt pressed film (blue) and the commercial suture (red) before exposure (solid line) and after exposure (dashed line) for 27 days to a pH 10 hydrolytic medium at 55 °C. (**b**) DSC heating runs of commercial sutures exposed for the indicated days to a pH 10 medium at 55 °C. (**c**) DSC heating runs of commercial sutures exposed at a pH 10 medium for 27 days and temperatures of 37 and 55 °C. For the sake of completeness, the curve for the commercial suture is also drawn. (**d**) DSC heating runs of commercial sutures exposed at pH 3, pH 7, and pH 10 media for 27 days and 55 °C.

Figure 3a shows the significant difference on the melting behavior between conventional melt pressed films and annealed sutures. Note that fusion is characterized by a predominant peak and a shoulder at a lower temperature (e.g., 49.7 and 58.2 °C for the film and 61.9 and 72.0 °C for the suture, heating rate of 10 °C/min), which reflects the existence of two populations of lamellar crystals with different thicknesses. The shoulder temperature strongly depends on crystallization and annealing processes since it is related to the less perfect formed crystals that are susceptible to reorganization processes. Therefore, molecular folds in these thinner lamellae underwent a slight reordering that led to an increase of the lamellar crystalline thickness. Basically, a simple melt crystallization leads to lamellae that are worse (i.e., lower thickness and more irregular folding surface) than those attained after annealing. Both shoulder and main melting peak logically appear at lower temperatures for the melt crystallized samples. It merits also attention the low value of the main melting peak, which indicates a limited reordering process of folds that precludes to get the highly organized lamellae derived from annealing (i.e., 58.2 °C with respect to 72 °C). Note also that the expected maximum melting temperature is reported to be 79.9 °C [20] as estimated from the Hoffman-Weeks extrapolation [25] for an infinite dimension of P4HB crystals.

Figure 3a also depicts the melting behavior of samples exposed to aggressive hydrolytic conditions (i.e., pH 10, temperature of 55 °C, and 27 days of exposure). Both types of samples, film and suture, shows the disappearance of the shoulder and a clear increase of the melting peak temperature. Degradation affects the folding surface, facilitates the reordering process, and leads to improved lamellae with a higher melting point. Note the difference around 7 °C that indicates the greater facility of annealed samples to render practically perfect crystals and that in this case a maximum melting temperature (79.0 °C) close to the equilibrium temperature was attained. Note also that molecular weight measurements showed only a moderate decrease during degradation, which means that thermal behavior is still associated to polymeric samples. Moreover, crystalline phases are those less susceptible to degradation and therefore should show lower changes on their associated properties (i.e., melting point).

The influence of the degradation time on the melting point is displayed in Figure 3b for the high temperature and the less pH aggressive conditions. A progressive increase of the melting point with the exposure time is clearly detected (i.e., from 74.1 to 79.0 °C for three and 27 days, respectively), as well as an increase of the melting enthalpy (i.e., from 57.8 to 63.3 J/g). The observation demonstrates that the crystalline lamellar thickness increases during degradation probably because of some chain breakages in the amorphous lamellar folding surfaces.

The increased chain mobility in the lamellar surface may favor the molecular reordering that leads to an increased crystalline lamellar thickness. In addition, an annealing effect caused, by the exposure to a degradation medium at 55 °C, may be discarded since the observed dependence with long exposure times is not well justified. A highlight also the fact that any stress that could favor annealing was not applied during degradation. Obviously, chain mobility is increased at 55 °C and the reordering process that took place after the chain breakage should be enhanced. In fact, degradation performed at 37 °C showed reasonably a less significant change.

Figure 3c compares the DSC curves of sutures exposed at pH 10 for 27 days at 37 and 55 °C. The sample exposed to the low temperature showed minor changes with respect to the initial suture that mainly affected the low temperature shoulder related to crystals more susceptible to reorganization (i.e., the temperature increased from 61.9 to 63.7 °C). In this case, the molecular weight decrease was low and the observed impact on thermal properties was limited to the preliminary phase concerning the less perfect crystals.

The impact of the pH of the medium on thermal properties was relatively scarce and the same kind of crystals seems to be attained at 55 °C after 27 days of exposure (Figure 3d). These correspond to the best reorganization that could be obtained from the initial commercial suture. Figure 3d confirms that temperature has a great influence on the degradation of the less perfect crystals since the peak shoulder completely disappeared. Table 1 summarizes the calorimetric data attained with representative samples.

**Figure 4:**
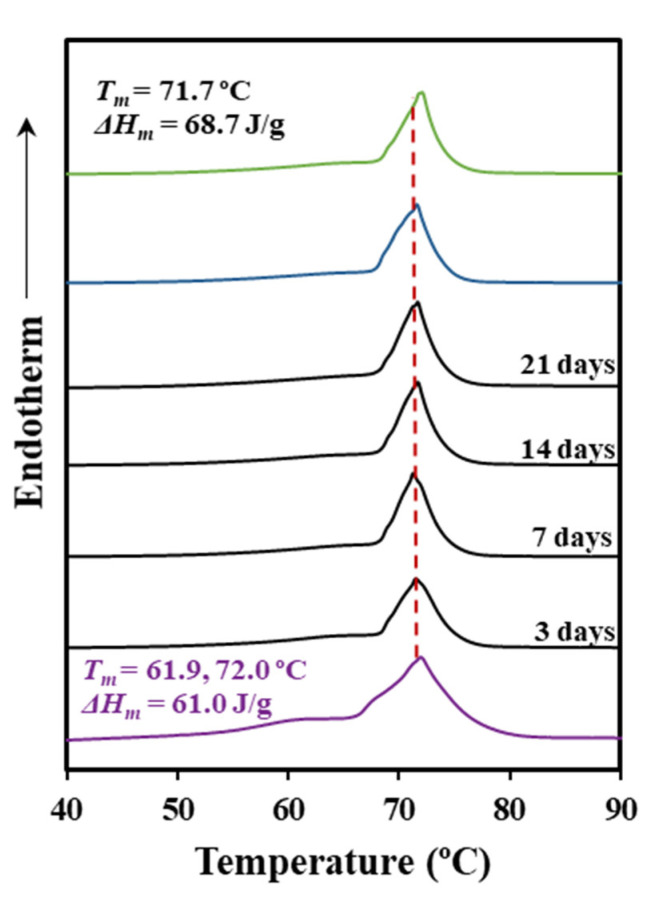
DSC heating scans (10 °C/min) corresponding to the initial suture (purple line), the suture exposed to a *Rhizopus oryzae* medium at 37 °C for the indicated days (black lines), the suture exposed to a *Pseudomonas cepacia* medium at 37 °C for 21 days (blue line), and the control (green line) (pH 7.4 medium for 21 days and 37 °C).

Thermal properties were scarcely affected by the enzymatic degradation. Thus, DSC curves for the control (hydrolytic medium without enzyme) and the two selected enzymatic media were practically identical (Figure 4, Table 2). The result agrees with an enzymatic surface erosion of the suture with significant loss of material that contrasts with the indicated bulk hydrolytic degradation mechanism.

**Figure 5:**
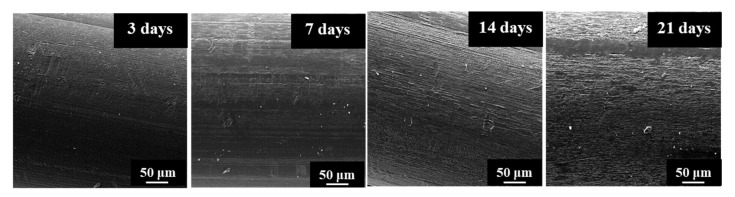
SEM micrographs showing the progressive surface erosion of P4HB sutures exposed to the *Rhizopus oryzae* medium at 37 °C for 3, 7, 14, and 21 days.

Figure 5 shows SEM micrographs that revealed an enzymatic attack that only affected the monofilament surface in a time dependent manner. Therefore, the DSC traces only reflect the impact of the hydrolytic degradation that as discussed before, mainly concerns the peak shoulder that decreased on intensity and moved from 61.9 to 64.3 °C.

### 3.3. Changes on Lamellar Microstructure during Degradation

The degradation behavior of highly annealed sutures is significantly different than observed for melt pressed films as a consequence of the different internal morphology. Stacking of oriented microfibrils with a lamellar organization and disordered spherulitic growth up to collision, are the specific morphologic trends of sutures and films, respectively. The impact of such morphologies led to a lower degradability of annealed samples due to their higher crystallinity. Furthermore, during degradation both crystallinity and melting temperature increased, although the effect was less significant for the annealed sample due to its scarce marge of improvement.

All studied P4HB samples displayed clear SAXS reflections that were analyzed to improve the comprehension of differences related to the supramolecular order since specific data concerning the geometrical parameters of constitutive lamellar structures could be easily derived.

Specifically, the study was performed from an isotropic integration of the oriented suture patterns or analyzing directly the disordered rings of film samples. In both cases, the normalized one-dimensional correlation function [26] was employed:

(2) $$\gamma \;(r)\;=\;\int_{0}^{\infty }q^{2}I(q)\cos(qr)dq/\int_{0}^{\infty }q^{2}I(q)dq$$

where *I* (*q*) is the intensity of the SAXS peak at each value of the scattering vector (*q* = [4π sin *θ*/λ] = 2π/*d*, with *θ* and *d* being the Bragg angle and the Bragg spacing, respectively). Basically, it is assumed that the lamellar stack is constituted by a high number of lamellae that had an infinite lateral size so the stack can be reduced to a one-dimensional two-phase structure that satisfies the Bragg condition.

Limited experimental collection of SAXS data was solved by extrapolation for low and high *q* values through the Vonk model [27] and the Porod’s law, respectively.

Analysis of the correlation function allows determining: (1) The long period, *L_γ_*; (2) the crystallinity within the lamellar stacks, *X*_c_^SAXS^; (3) the crystalline lamellar thickness, *l*_c_, and the amorphous layer thickness, *l*_a._ In this way, *L_γ_* corresponds to the *r* value of the first maximum of the correlation function; *l*_a_ has been assigned to the *r* value for the intersection of the LRAT (linear regression in the autocorrelation triangle) with the ordinate equal to the first minimum of the correlation function; *l*_c_ corresponds to *L_γ_*, *l*_a_; and *X*_c_^SAXS^ is calculated as *l*_c_/*L_γ_*. The lower thickness of the two-phase lamellar model has been assigned to the amorphous layer thickness although the correlation function cannot distinguish the thickness associated with each phase.

**Figure 6:**
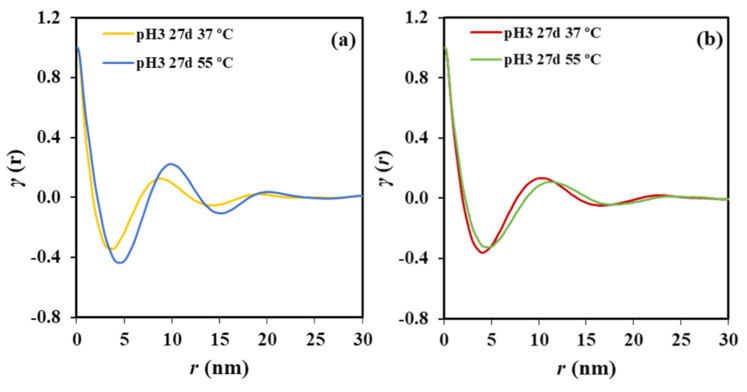
(**a**) Correlation function of the SAXS peak determined for the P4HB film exposed to a pH 3 medium at 37 and 55 °C for 27 days. (**b**) Correlation function of the SAXS peak determined for the P4HB suture exposed to a pH 3 medium at 37 and 55 °C for 27 days.

Figure 6 illustrates representative correlation functions that allows comparing and inferring a distinct evolution of films and sutures during the hydrolytic degradation. Thus, the progression of film degradation led to a shift of the correlation function to higher distances and also to more pronounced peaks. Therefore, *L_γ_* increased from 8.60 to 9.80 nm when the temperature of the hydrolytic medium increased from 37 to 55 °C, a change that was a consequence of the increase of the lamellar crystalline thickness (i.e., *l_c_* increased from 6.91 to 7.32 nm). Moreover, an increase was also observed for the amorphous layer thickness (i.e., from 1.69 to 2.48 nm) leading to a practically constant crystallinity of the lamellar stack (i.e., 80 ± 1%). The amorphous phase seems to be less dense due to the increasing thickness probably caused by the chain breakage. The increase on the electronic contrast between crystalline and amorphous phases is observed through the more pronounced profile of the correlation function.

Figure 6b displays by contrast that the correlation profile becomes smoother when degradation increases, a feature that cannot be well explained at this stage and a more accurate evaluation of the microstructure of the biphasic systems is required. Nevertheless, a slight shift of the correlation function to the increasing distances is clear. Specifically, *L_γ_* and *l_c_* increased from 10.30 and 8.31 nm to 10.90 and 8.80 nm, respectively when the temperature of the degradation medium increased from 37 to 55 °C. Underlined here that changes are moderate due to the high initial thickness of the annealed lamellae as previously deduced from the closeness between the experimental melting temperature and the theoretical value deduced from the equilibrium melting temperature. Furthermore, the crystallinity of the lamellar stack remained equal to 80.7%.

In addition to the *L_γ_* value, which is associated with the most probable distance between the centers of gravity of two adjacent crystals, a long period determined from twice the value of the first minimum of the correlation function, $L_{\gamma }^{m}$, is also useful. This is interpreted as the most probable distance between the centers of gravity of a crystal and its adjacent amorphous layer. A discrepancy between both values indicates a broad distribution of the layer widths of the major component [28], which in this case corresponds to the crystal phase.

Table 3 summarizes the morphological parameters determined for representative degraded film and suture samples. The following trends can be indicated: (a) Discrepancy between *L_γ_* and 2 × *L_γ_^m^* is decreasing as the degradation process becomes more significant. This feature can be explained considering the lamellar reordering process that, for example, lead to a decrease of the population of thinner crystals in film samples, and consequently to a narrow distribution. Note, for example, that differences around ~1 and ~0.6 nm are determined for degradations performed at 37 and 55 °C. (b) Annealed samples showed a greater discrepancy than films (e.g., ~1 and ~2.1 nm for films and sutures, respectively). This feature seems strange since a narrow distribution is expected for the thicker annealed lamellae of sutures.

**Figure 7:**
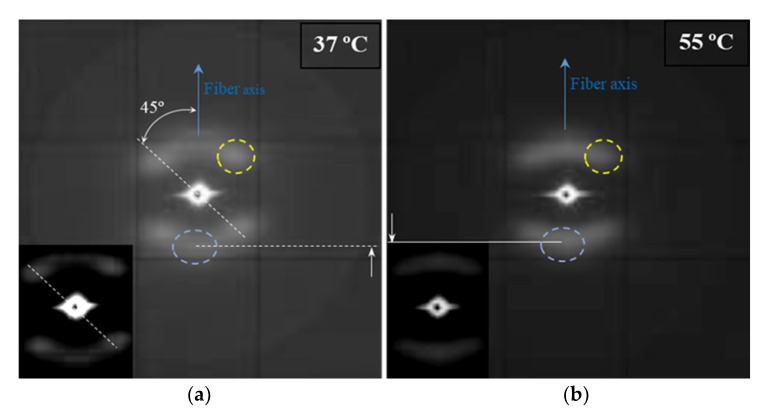
The SAXS patterns of an annealed suture submitted to low (i.e., pH 10, 37 °C, 27 days) (**a**) and high (i.e., pH 3, 55 °C, 27 days) (**b**) degradation processes. Insets show low contrast exposures of the corresponding patterns. Meridional and off meridional reflections are indicated by the blue and yellow dashed circles, respectively. Solid and dashed white lines highlight the different positions of meridional reflections.

The SAXS pattern displayed in Figure 7a for a sample exposed to a very little aggressive degradation condition (therefore similar to that observed with the initial suture) reveals that the thermal annealing process at which the commercial sutures were submitted lead to a peculiar morphology where two different types of lamellar stacks exists. These differences come up from the distinct lamellar organization in the skin and the core of sutures. Obviously, this phenomenon is of a different nature than that caused by a simple crystallization process, where usually populations of lamellae with different thicknesses and organizations of folding surfaces are derived. The observations justify the above indicated broad lamellar distribution found in sutures.

The SAXS pattern is characterized by four off meridional spots and two meridional arches (Figure 7a). The first ones are indicative of the stacking of breadth lamellar crystals tilted with respect to the fiber direction and logically arranged with a cylindrical symmetry. The characteristic spacing of these stacks is 14.0 nm. The second ones have a higher spacing (i.e., 14.1 nm) and corresponds to lamellar crystals with lower lateral extension (longer and diffuse reflection) and perpendicularly oriented to the fiber axis. The nanostructure of the core material as compared to the shell material appears rougher and more imperfect. The external part of sutures is submitted to a higher temperature than the core and suffers a higher shear stress. In this way, a shift between molecular chains along the annealing direction of lamellae, as well as an increase of the lamellar thickness is produced. Therefore, tilted lamellar surfaces, which moreover appeared at an angle of 45° that correspond to the maximum shear, are generated.

The microstructure of sutures changed during hydrolytic degradation as can be deduced from the SAXS pattern (Figure 7b) of the sample exposed to the more aggressive conditions (i.e., pH3, 55 °C, and 27 days). Basically, differences concerning the meridional spots associated to the more imperfect crystals are placed in the core. Thus, the interlamellar spacing slightly increased (from 14.1 to 14.8 nm) as the reflections slightly moved to the center of the pattern. Furthermore, the intensity of these spots increased suggesting a higher electronic contrast between crystalline and amorphous layers. In this way, degradation mainly affected the more defective crystals, causing some molecular breakages on their folding surface. A slight reordering was produced leading to the observed increase of the crystalline lamellar thickness, while simultaneously the amorphous layer became less compact and more disordered.

**Figure 8:**
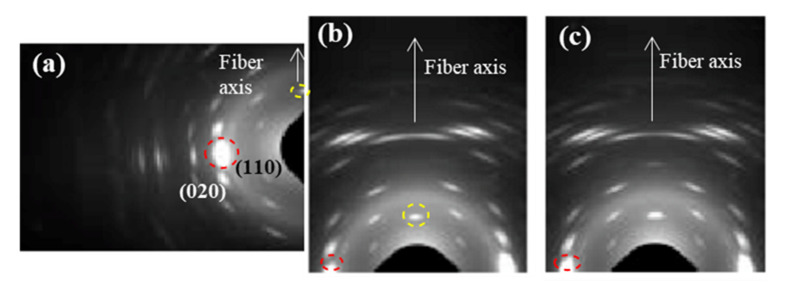
WAXD patterns of an annealed suture submitted to low (i.e., pH 10, 37 °C, 27 days) (**a**,**b**) and high (i.e., pH 3, 55 °C, 27 days) (**c**) degradation processes. Only a region of the reciprocal space is registered due to the specific configuration of the beamline that allows recording simultaneously SAXS and WAXD patterns. (**a**,**b**) Patterns were obtained from different orientations of the suture in the holder in order to get information of both equatorial (**a**) and meridional (**b**) reflections. Common meridional and equatorial reflections are indicated by the dashed yellow and red circles.

Molecular chains in the crystalline lamellae remained aligned with the longitudinal direction of sutures, even those crystals with tilted surfaces. All observed reflections in WAXD patterns (Figure 8) were in agreement with the published orthorhombic unit cell of P4HB [16,17]. Specifically, (110) and (020) reflections at 0.388 and 0.406 nm appeared as very small arcs in the equator.

**Figure 9:**
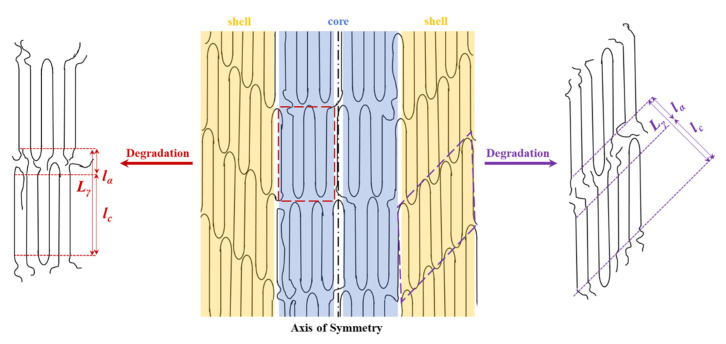
Scheme showing the structure of annealed fibers characterized by the presence of two types of lamellar crystals that are part of the shell and the core before and after being submitted to hydrolytic degradation.

The scheme of Figure 9 illustrates the deduced microstructure of the annealed fiber and the consequences of the hydrolytic attack. The lamellar thickness remained unaltered for the more perfect tilted crystals and even a slight densification was detected for their stretched folds, since the intensity of the corresponding spots seemed to decrease. In fact, this deduction is in agreement with the previously indicated contradictory results determined from the analysis of the correlation function. The crystalline structure remained unchanged, as well as the degree of orientation of crystals since no change was detected in the WAXD patterns as displayed in Figure 8c.

**Figure 10:**
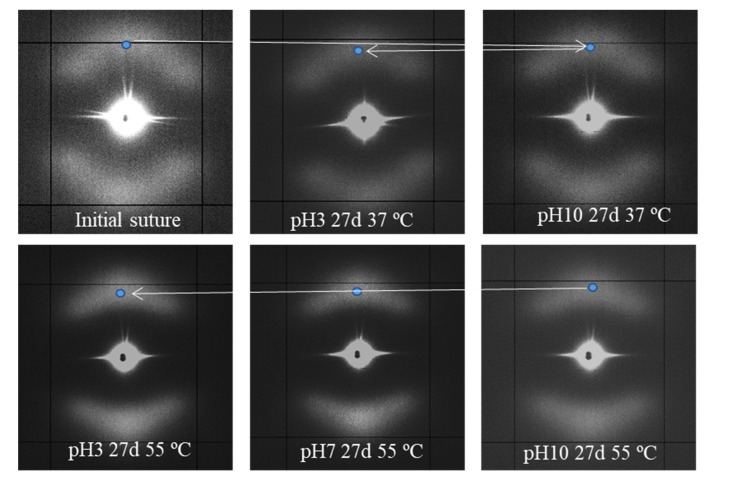
SAXS patterns of the initial suture and those exposed to the indicated hydrolytic conditions.

Intensification of the meridional spot and its shift to the center of the pattern shows a correlation with the degradation degree, as can be inferred from the gradual evolution (see blue spot) presented in Figure 10 for representative conditions.

**Figure 11:**
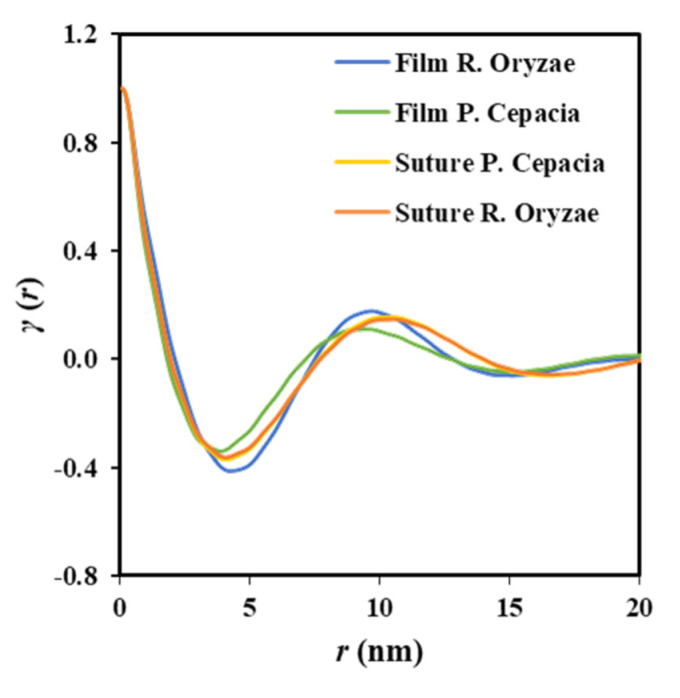
Correlation function of the SAXS peak determined for P4HB films and sutures exposed to *Pseudomonas cepacia* and *Rhizopus oryzae* media at 37 °C for 14 days.

Despite the fact that enzymatic degradation was effective as deduced from a weight loss of 8–9% after 21 days of exposure to both assayed media (Figure 2a), the impact on the microstructure of the remaining material should be minimum as reflected by the scarce change on the molecular weight (Figure 2b) and the melting point (Figure 4 and Table 2). This is corroborated through analysis of SAXS patterns (Figure 11), since no changes were detected between samples exposed to the less (i.e., *Pseudomonas cepacia*) and the more (i.e., *Rhizopus oryzae*) aggressive media for 14 days. Thus, *L_γ_*, *l_c_*, *l_a_*, and $L_{\gamma }^{m}$ parameters remained practically constant and equal to 10.40–10.50, 8.33–8.56, 2.07–1.94, and 4.1 nm (Table 3), respectively. Note again the high discrepancy between *L_γ_* and 2 × $L_{\gamma }^{m}$ values as expected from the existence of two well differentiated types of lamellae. Figure 11 also shows the clearly different susceptibility to the enzymatic attack of P4HB films constituted by spherulitic morphologies. In this case, a clear increase of lamellar spacing was detected (Table 3), as well as on the electronic contrast. Logically, differences came from the different degradability of films and sutures, with the weight loss of the former being for example around (75–95%).

**Figure 12:**
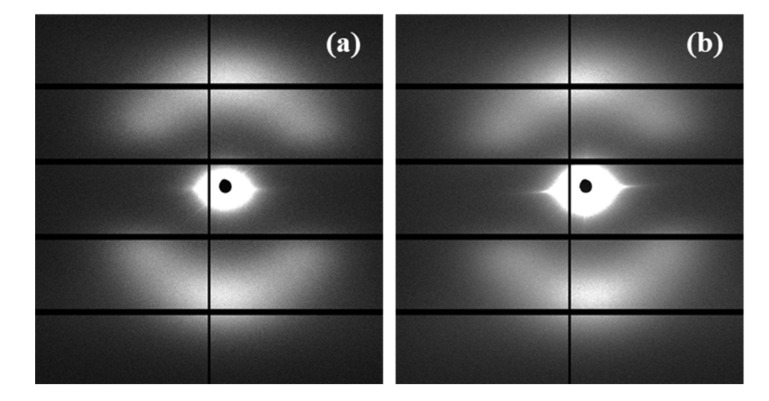
SAXS patterns of suture exposed to *Pseudomonas cepacia* (**a**) and *Rhizopus oryzae* (**b**) media at 37 °C for 14 days.

SAXS patterns of the less and more degraded sutures were again highly similar, considering both the position (angle and distance) of the observed spots and the relative intensity between meridional and off-meridional reflections (Figure 12). In conclusion, there are no evidences of the observed morphological change that occurs at an advanced stage of hydrolytic degradation. These results are fully consistent with an enzymatic surface attack that led to an erosion of the suture (see Figure 5) and did not change the internal microstructure of the remaining material.

### 3.4. Changes on Lamelar Microstructure of Degraded Samples during Heating

The evolution of SAXS patterns during heating processes can give relevant information concerning the microstructure of sutures as detected, for example, with segmented glycolide based copolymers. These exhibited clearly differentiated behaviors depending on the degradation treatment [20]. Annealed P4HB sutures showed less changes during heating due to the high perfection of crystals, the more reduced presence of intralamellar amorphous regions and the lack of any evidence related to the presence of regularly distributed interfibrilar amorphous regions. These should be originated from disordered regions placed on lateral sides of lamellae arranged in a fibrillar way and should lead to patterns with equatorial reflections.

**Figure 13:**
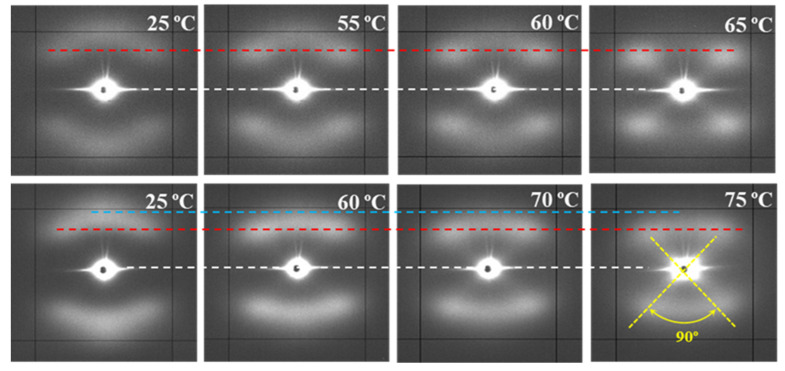
SAXS patterns of P4HB sutures exposed to hydrolytic media of pH 10, at 37 °C (top row) and pH 3, at 55 °C (bottom row) for 27 days. Patterns were recorded at the indicated temperatures during a heating run performed at 10 °C/min. Dashed lines are used as references for the position of the center of the pattern (white), off-meridional spots (red), and meridional spots (blue).

Figure 13 compares the temperature evolution of patterns of representative samples hydrolytically degraded at 37 and 55 °C, with the previously indicated differences being highlighted.

The following features can be indicated: (a) Lamellae that constitute the core of the suture are more imperfect and therefore initiates melting at lower temperatures than the tilted ones. A continuous decrease on their intensity is detected when temperature approaches the melting of the suture, while the intensity of tilted lamellae remained practically constant. It is interesting to point out that the suture degraded at 37 °C and heated to 55 °C still demonstrates weaker meridional spots, allowing to discard that a thermal annealing process could intensify the meridional spots in the case of degradation performed at 55 °C. (b) Meridional reflections can still be envisaged at temperatures very close to the fusion of the suture for the more degraded sample, while they practically disappeared for the samples exposed to the 37 °C medium. (c) Lamellar spacings increase with temperature for both tilted and non-tilted lamellae, although the effect is clearer for the last ones (Table 4). (d) Tilted surfaces have always the same orientation with respect to the meridian (i.e., ±45°).

## 4. Conclusions

Stretched P4HB commercial sutures were characterized by two different types of lamellar stacks. These were originated by the different shear stress that experimented the shell and the core of the fibers during processing. Therefore, the stacks placed in the shell were constituted by the thicker lamellae having tilted surfaces as a consequence of the slippage of molecular chains. The inner stacks were constituted by more imperfect lamellae having surfaces perpendicularly oriented to the fiber axis. SAXS patterns revealed the interlamellar spacings but did not show any evidence concerning the possible existence of interfibrillar amorphous regions.

Different degrees of degradation could be achieved by exposure to hydrolytic media by modifying pH, temperature, and time. Significant chain breakages were found from GPC measurements, but weight losses were practically depreciable even under the most aggressive conditions. Microstructural changes were found dependent on the progress of degradation and distinctly affected the lamellar stacks. More significant changes were observed for the inner lamellar that experimented a higher increase of both crystalline and amorphous lamellar thicknesses. Hence, the chain breakages occurred in the amorphous regions of a more disordered surface and allowed a certain reorganization of the chains which increased the crystalline region. On the contrary, enzymatic degradation only caused a surface erosion with loss of surface material and had a scarce influence on the microstructure.

Both hydrolytic and enzymatic degradation were different for melt crystallized films. In this case, the microstructure was characterized by spherulites constituted by thinner lamellae with a relatively narrow distribution. The progress of degradation caused a significant thickening that affected the thermal properties.

Heating of stretched sutures revealed again differences according to the different degrees of degradation experimented by the distinct types of lamellar stacks. Thus, a slight thickening was detected for both types of lamellae as a consequence of the typical temperature reordering process, but with the melting of lamellae placed in the fiber core being clearly faster.

## Figures and Tables

**Table 1:** Melting peak temperatures and enthalpies of P4HB sutures degraded at the indicated pH values, temperatures, and exposure times.

Samples	*T_m_* (°C)	*ΔH_m_* (J/g)
pH10 27d 37 °C	63.7, 70.7	67.0
pH3 27d 55 °C	79.0	63.3
pH7 27d 55 °C	79.0	63.3
pH10 3d 55 °C	74.1	57.8
pH10 7d 55 °C	75.0	58.2
pH10 14d 55 °C	76.8	60.9
pH10 27d 55 °C	79.0	63.3

**Table 2:** Melting peak temperatures and enthalpies of P4HB sutures exposed at 37 °C to the indicated enzymatic degradation medium and exposure time.

Enzymes	Time (days)	*T_m_* (°C)	*ΔH_m_* (J/g)
Control	21	72.1	68.9
*P. cepacia*	3	71.4	67.3
*P. cepacia*	21	71.6	68.0
*R. oryzae*	3	71.5	68.1
*R. oryzae*	21	71.7	68.7

**Table 3:** Morphological parameters of films and sutures exposed to degradation at the indicated media.

Sample	*L_γ_* (nm)	*l_c_* (nm)	*l_α_* (nm)	$L_{\gamma }^{m}\;(\mathrm{nm})$
Film pH3 27d 37 °C	8.60	6.91	1.69	3.8
Film pH10 27d 37 °C	8.50	6.88	1.62	3.2
Film pH3 27d 55 °C	9.80	7.32	2.48	4.6
Film pH10 27d 55 °C	9.6	7.62	1.98	4.1
Suture pH3 27d 37 °C	10.30	8.31	1.99	4.1
Suture pH10 27d 37 °C	9.80	7.91	1.89	4.0
Suture pH3 27d 55 °C	10.90	8.80	2.10	4.8
Suture pH10 27d 55 °C	10.70	8.66	2.04	4.3
Film *R. oryzae* 14 days 37 °C	9.60	7.25	2.35	4.3
Film *P. cepacia* 14 days 37 °C	9.50	7.72	1.78	3.9
Suture *R. oryzae* 14 days 37 °C	10.50	8.56	1.94	4.1
Suture *P. cepacia* 14 days 37 °C	10.40	8.33	2.07	4.1

**Table 4:** Spacings of the reflections observed in SAXS patterns recorded at different temperatures during heating of hydrolytically degraded samples (pH 10, 27 days) at 37 °C and (pH 3, 27 days) 55 °C.

Degradation Temperature (°C)	Temperature (°C)	*L_meridional_*^(a)^ (nm)	*L_off-meridional_*^(a)^ (nm)
37	25	14.1	14.0
37	55	14.9	14.3
37	60	15.9	14.9
37	65	16.1	15.3
55	25	14.8	14.6
55	60	16.9	16.0
55	70	18.5	16.4
55	75	20.8	17.4

^(a)^ Spacings directly measured on the pattern are always slightly higher than *L_γ_* values evaluated with the correlation function.
